# The effects of previous abdominal surgery and the utilisation of modified access techniques on the operative difficulty and outcomes of laparoscopic cholecystectomy and bile duct exploration

**DOI:** 10.1007/s00464-024-10949-x

**Published:** 2024-07-01

**Authors:** James Lucocq, Ahmad H. M. Nassar

**Affiliations:** 1 Department of Surgery, Ninewells Hospital and Medical School, Dundee, Scotland, UK; 2 Laparoscopic Biliary Surgery Unit, University Hospital Monklands, Lanarkshire, Scotland; 3 University of Glasgow, Glasgow, Scotland, UK; 4 Golden Jubilee National Hospital, Glasgow, Scotland

**Keywords:** Laparoscopic cholecystectomy, Previous abdominal surgery, Abdominal scars, Adhesiolysis, Modified access, Complications, Difficulty grading

## Abstract

**Background:**

Early reports suggested that previous abdominal surgery was a relative contraindication to laparoscopic cholecystectomy (LC) on account of difficulty and potential access complications. This study analyses different types/systems of previous surgery and locations of scars and how they affect access difficulties. As modified access techniques to minimise risk of complications are under-reported the study details and evaluates them.

**Method:**

Prospectively collected data from consecutive LC and common bile duct explorations (LCBDE) performed by a single surgeon over 30 years was analysed. Previous abdominal surgery was documented and peri-operative outcomes were compared with patients who had no previous surgery using Chi-squared analysis.

**Results:**

Of 5916 LC and LCBDE, 1846 patients (31.2%) had previous abdominal surgery. The median age was 60 years. Those with previous surgery required more frequent duodenal (RR 1.07; *p* = 0.023), hepatic flexure (RR 1.11; *p* = 0.043) and distal adhesiolysis (RR 3.57; *p* < 0.001) and had more access related bowel injuries (0.4% vs. 0.0%; *p* < 0.001). Previous upper gastrointestinal and biliary surgery had the highest rates of adhesiolysis (76.3%), difficult cystic pedicles (58.8%), fundus-first approach (7.2%), difficulty grades (64.9% Grades 3–5) and utilisation of abdominal drains (71.1%). Previous open surgery resulted in longer operative time compared to previous laparoscopic procedures (65vs.55 min; *p* < 0.001), increased difficulty of pedicle dissection (42.4% vs. 36.0%; *p* < 0.05) and required more duodenal, hepatic flexure and distant adhesiolysis (*p* < 0.05) and fundus-first dissection (4% vs 2%; *p* < 0.05). Epigastric and supraumbilical access and access through umbilical and other hernias were used in 163 patients (8.8%) with no bowel complications.

**Conclusion:**

The risks of access and adhesiolysis in patients with previous abdominal scars undergoing biliary surgery are dependent on the nature of previous surgery. Previous open, upper gastrointestinal and biliary surgery carried the most significant risks. Modified access techniques can be adopted to safely mitigate these risks.

**Graphical abstract:**

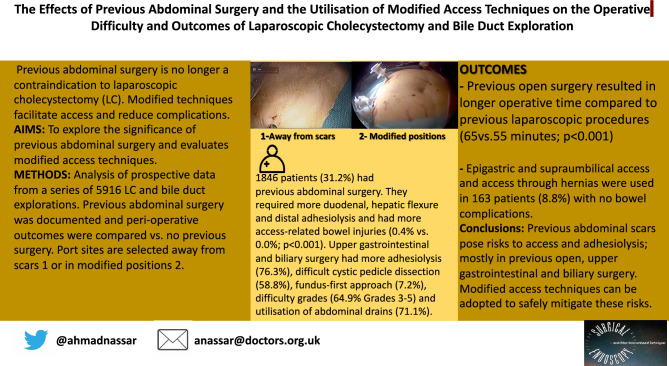

Previous abdominal surgery involving certain procedures was considered, in some early reports, a relative contraindication to LC because of a perceived increase in the difficulty of establishing pneumoperitoneum and of dealing with adhesions to access the gallbladder, subsequently increasing the risk of complications. The possibility of adhesions between bowel, omentum or liver to the anterior abdominal wall increases the risk of trocar-related injury on entry [1]. This remains the case whether closed or open access methods are used. Adhesions may also hinder access to the gallbladder or the whole of the right upper quadrant, reduce visualisation of the gallbladder or the cystic pedicle and increase the difficulty of dissection, risk of complications and incidence of open conversion [2].

Although multiple studies have reported outcomes following LC in patients with previous abdominal surgery, the literature lacks a detailed analysis across a large patient cohort of multiple previous operation types [3–7]. Moreover, modified access techniques that facilitate access and dissection and reduce the risk of complications are under-reported.

The primary aim of this study was to explore the significance of previous abdominal surgery and the location of the resulting scars in patients undergoing LC and LCBDE and to evaluate various modifications of the access technique. The secondary aim was to study how they affected the operative difficulty, surgery time, operative complications and conversion rates.

## Methods

Analysis of data from consecutive patients undergoing LC and LCBDE performed under the care of one surgeon between 1992 and 2021. The surgeon set up a laparoscopic service after a short laparoscopic surgery course, as he had previously completed surgical training in the open surgery era and subsequently established a referral firm specialising in index admission surgery for all comers with biliary emergencies and single session management of those with suspected bile duct stones. All procedures in this study were performed by the senior author or by his trainees under direct on-table supervision. He received all complex biliary cases while elective cholecystectomies operated on by other surgeons for intermittent periods at the same centre were not part of this study.

### Data collection and analysis

Prospectively collected data from 5916 patients, including patient demographics, type of admission, clinical presentation, radiological findings, operative findings, operative difficulty grade, details of technique and peri-operative complications were analysed. Throughout the study, there was an intention to treat all patients laparoscopically. Therefore, all patients undergoing laparoscopic cholecystectomy or bile duct exploration were included in the analysis. None of the patients in the whole cohort were preselected for open cholecystectomy during the prospective phase or excluded from analysis for the purpose of this study based on having abdominal scars resulting from previous abdominal surgery. The speciality of the previous abdominal operation, type of surgery (open or laparoscopic), the site/s of abdominal scars and the use of modified access techniques were documented. Groin hernia repairs were not recorded as, unlike upper and lower abdominal surgery, they do not pose a challenge for the purpose of laparoscopic access.

The outcomes of patients with and without previous surgery were compared using *χ*^2^ analysis. A* p* value of < 0.05 was considered statistically significant. All analyses were performed using R Studio 2022.02.02.

Informed consent was obtained from all patients with emphasis on the potential need to change the access method and on the risk of complications specific to access and adhesiolysis. The database was registered as a clinical audit and no ethical approval was necessary as this was a retrospective analysis of a clinical study using a standard protocol for LC according to national and international guidelines.

This study was conducted according to the STROBE Guidelines [8].

### Operative technique

#### Closed access

During the first five years of the series access was achieved by the closed method; inserting a Verres needle either below or above the umbilicus, performing the drop test and inserting a sharp trocar and cannula for the first port. The needle was inserted on the opposite side to any abdominal scars. Occasional access was achieved by placing a trocar through the defect of a pre-existing umbilical hernia or a right subcostal point on only two occasions.

#### Open access

The first “camera” port was established by open access for the last 24 years. A transverse incision is made just below the umbilicus and the subcutaneous fat cleared to expose the junction of the umbilical tube and the fascia. The umbilical tube is lifted with an artery forceps and the fascia is held with a Littlewood grasper. A small incision is made at the junction between the umbilical tube and the fascia using scissors or a knife. A blunt Mayo artery forceps is used to enter that incision and into the peritoneal cavity, directed towards the right upper quadrant at 45° in relation to the upper midline and entering the peritoneum at 30° to the abdominal wall. Once the tip of the forceps is in the peritoneal cavity it is withdrawn to the fascia and used to stretch it to 10 mm before being removed. A blunt cannula is then inserted through the fascial incision into the peritoneal cavity and a laparoscope is inserted to confirm the intraperitoneal position as the cannula is connected to CO_2_ and the pneumoperitoneum is established. The tight fitting of the cannula into the fascial incision usually prevents gas leakage. With this access technique the use of sutures or balloon cannulas was seldom necessary. Careful inspection of the area under the umbilicus is carried out to ensure the integrity of the omentum and any underlying bowel. Attention is then directed to inserting the secondary ports under vision. When scars of previous abdominal surgery are encountered this may occasionally need to be facilitated by sweeping filmy adhesions with the tip of the telescope to clear the parietal peritoneum at the site of an intended secondary port. The following options for access were considered and used where appropriate, subject to the encountered scars:

1. Scars of previous laparoscopic access at the umbilicus, right subcostal or “roof top” scars: no modifications of the above technique were necessary where periumbilical laparoscopic port site scars were encountered e.g. laparoscopic sterilisation, laparoscopic appendicectomy etc. The primary infraumbilical access site is usually uncomplicated in the presence of scars at the above positions and did not encounter, in this study, any significant adhesions of omentum or bowel to the parietal peritoneum at the umbilicus.
2. Lower midline scars are best avoided by positioning the camera port above the umbilicus, using the above technique, whether or not the scar reaches it.
3. Upper or full length midline scars are avoided by making an incision 2–3 cm below the xiphisternum, incising the fascia between two Littlewood graspers and entering the peritoneal cavity at 30° towards the costal margin to avoid any adhesions. The 12 mm cannula is inserted with the telescope to confirm intraperitoneal positioning before establishing pneumoperitoneum. This allows the cannula to avoid the liver or to redirect it should it enter the falciform ligament. A second 5 mm port is inserted under vision where the parietal peritoneum is clear of adhesions and is used to start adhesiolysis to clear the sites selected for further ports. These sites will be dictated by the ease of sweeping adhesions. Where small bowel loops are closely adherent to the parietal peritoneum their mobilisation should be avoided, modifying the positions of the secondary ports instead. A 5 mm camera port site may be placed in the right iliac fossa if the area underneath the umbilicus has dense adhesions [9].
4. Para-umbilical hernias, either primary or incisional, are usually used for access. The skin is incised over the defect which is displayed and then its edge entered with a blunt forceps as described above before placing the first cannula. The excision of a sac and the closure of the defect is carried out at the end of the procedure in the manner previously described [10]_._

Following intra-abdominal access, LC was performed using a standard four port technique with the patient in the American position. Adhesiolysis was performed when required and the division of adhesions (e.g. distal, hepatic flexure, duodenal and gallbladder) was documented. Once adhesions were swept away from the gallbladder the standard approach to the cystic pedicle was employed to identify the cystic duct and cystic artery. The peritoneum was opened, any fat was cleared and the identified structures filleted with blunt dissection using the “duckbill” grasper. This study did not use hook diathermy after the first few cases, in favour of blunt grasper dissection and swab dissection in the presence of inflammation. Alternating posterior and anterior dissection was carried out whilst maintaining the dissection lateral to the cystic lymph node, if identified, as a guide to the cystic artery until the gallbladder neck was identified and encircled and the cystic artery was encircled as it entered the gallbladder wall lateral to the cystic lymph node. This infundibular approach was used in the first half of the series. Following this the critical view of safety was achieved routinely, whenever possible, by confirming the two structures entering the gallbladder wall and clarifying the nature of any other structures reaching the gallbladder once exposing the proximal third of the hepatocystic plate was done. Occasional additional structures are then opened, small vessels can be cauterised and any other structures that do not appear to be vessels are reassessed, confirmation of their entry into the gallbladder obtained, documented through images and/or video and are treated as ductal structures to be ligated as hepatocystic ducts. The two main structures are only secured once the anterior and posterior windows were confirmed.

The difficulty of the procedure was assessed prospectively using the Nassar grading system [11]. Intraoperative cholangiography was routinely attempted.

## Results

5916 consecutive patients undergoing LC were included in the study with a median age of 51 years; a M:F ratio of 1:3, and a median ASA of 2. Subject to the clinical presentation one or more of the following admission diagnoses were recorded: chronic biliary pain in 3336 patients (56.4%), acute pain in 1876 patients (31.7%), obstructive jaundice with or without cholangitis in 1096 patients (18.5%), acute cholecystitis in 554 patients (9.4%) and acute pancreatitis in 464 patients (7.8%). 3221 patients (54.4%) had an elective cholecystectomy. The background characteristics of the entire cohort, including subgroups of previous and no previous abdominal surgery are reported in Table 1.

**Table 1:** Background data of patients with and without previous abdominal surgery undergoing laparoscopic cholecystectomy

Variable		All patients (*n* = 5916)	Previous abdominal surgery, *n* = 1846 (%)	No previous abdominal surgery, *n* = 4070 (%)	*p*-value (previous vs. no surgery)
Age, Years (%)	< 40	1509 (25.5)	347 (18.8)	1162 (28.6)	< 0.001
	40–59	2340 (39.6)	773 (41.9)	1567 (38.5)	
	≥ 60	2066 (34.9)	726 (39.3)	1340 (32.9)	
Male:female		1542:4373 73.9%	244:1602 86.8%	1298:2771 68%	< 0.001
American Society of Anaesthesiologists Score (%)	1	2051(34.7)	549 (29.7)	1502 (36.9)	< 0.001
	2	2444 (41.3)	815 (44.1)	1629 (40.0)	
	≥ 3	1410 (23.8)	482 (26.1)	928 (22.8)	
Pre-operative imaging	Ultrasound Abdomen	5619 (95.0)	1740 (94.3)	3879 (95.3)	< 0.001
	CT Abdomen/pelvis	223 (3.8)	58 (3.1)	165 (4.1)	0.088
	MRCP	332 (5.6)	94 (5.1)	238 (5.8)	0.24
Clinical presentation based on biochemical and radiological findings	Jaundice	314 (5.3)	78 (4.2)	236 (5.8)	0.01
	Acute cholecystitis	544 (9.2)	141 (7.6)	403 (9.9)	0.005
	Cholangitis	138 (2.3)	45 (2.4)	93 (2.3)	0.72
	Pancreatitis	464 (7.8)	128 (6.9)	336 (8.3)	0.08
Pre-operative ERCP		155 (2.6)	46 (2.5)	109 (2.6)	0.68

### Evolution of the access technique

Prior to April 1997, before moving to the use of open access techniques, closed Veress needle access was utilised in 356 patients. Of these 102 (28.6%) had had previous abdominal surgery resulting in scars in the right lower quadrant in 39 patients, umbilical or lower midline in 31, suprapubic in 23, right upper quadrant in 4 and upper abdomen in 1. The scars were not documented in four patients. Adhesiolysis to complete access and establish secondary ports was necessary in 16 patients and open conversion occurred in one patient where the first cannula had entered omental adhesions and a satisfactory view could not be obtained. In the last 24 years the remaining cohort underwent open access as described above.

### Previous abdominal surgery

Previous abdominal surgery had been performed in 1846 patients, 31.2% of the entire cohort (median age 60 years, median ASA, 2). Those who had undergone previous abdominal surgery were older (*p* < 0.001), had higher ASA (*p* < 0.001) and were more likely to be female (*p* < 0.001) than those who had no previous surgery (Table 1). 40% were emergency admissions and, in view of the specialist interest of this unit, 31.5% had risk factors for bile duct stones. 16.7% of the cholecystectomies and/or bile duct explorations were either referred to this unit from other hospitals or performed at other hospitals by the senior author.

Trainees were involved in 408 patients (22%) of the cohort with previous surgery, performing components of the procedures appropriate to their stage of training. They performed the whole procedure, including access, in 141 (34.5%) of these patients. All were patients not requiring modified access.

The requirement for division of adhesions between the gallbladder and either the duodenum (RR = 1.07; *p* = 0.023) or the hepatic flexure (RR 1.11; *p* = 0.043) increased slightly in patients who had previous surgery. However, the increase in distant adhesiolysis in such patients was significant (R 3.57; < 0.001). Overall, the Calot’s triangle dissection was more difficult (*p* < 0.001) in those without previous abdominal surgery as a result of the high rate of emergency admissions with acute clinical presentations, except in those with previous upper abdominal or biliary surgery. There was no significant difference in the difficulty grading of the cholecystectomy, the rate of abdominal drains, or the use of alternative surgical approaches (e.g. fundus-first dissection or open conversion) in those with previous abdominal surgery compared to those with no previous surgical history (Table 2). As this unit has a major interest in single session management of ductal stones via laparoscopic exploration of the bile duct, 380 patients (20.6%) with previous surgery had positive cholangiography and underwent bile duct exploration.

**Table 2:** Operative data in patients with or without scars of previous abdominal surgery

Variable		All patients, *n* = 5916 (%)	Previous surgery, *n* = 1846 (%)	No previous surgery, *n* = 4070 (%)	RR	*p*-value (previous vs. no surgery)
Adhesiolysis: gallbladder to	Omentum	3754 (63.5)	1193 (64.6))	2561 (62.9)	1.0	0.19
	Duodenum	2732 (46.2)	893 (48.3)	1839 (45.2)	1.1	0.02
	Hepatic flexure	1205 (30.4)	405 (21.9)	800 (19.7)	1.1	0.04
Distal adhesiolysis		770 (13.0)	475 (25.7)	295 (7.2)	3.6	< 0.001
Difficult cystic pedicle dissection		2362 (39.9)	676 (36.6)	1686 (41.4)	0.9	< 0.001
Nassar difficulty grading	I	1959 (33.1)	608 (32.9)	1351 (33.2)	1.0	0.85
	II	1771 (29.9)	571 (30.9)	1200 (29.5)	1.1	0.26
	III	1192 (20.1)	357 (19.3)	835 (20.5)	0.9	0.30
	IV	869 (14.7)	264 (14.3)	605 (14.9)	1.0	0.57
	V	124 (2.1)	46 (2.5)	78 (1.9)	1.3	0.15
Fundus-first dissection		179 (3.0)	60 (3.3)	119 (2.9)	1.1	0.50
Abdominal drain		3052 (51.6)	932 (50.5)	2120 (52.1)	1.0	0.25
Median operative time (min)		60	60	60	-	0.54
Conversion to open		28 (0.5)	5 (0.2)	23 (0.6)	0.3	0.13

A comparison between peri-operative outcome parameters in those with or without a history of previous surgery is shown on (Table 3). The only significant complications resulting from access or adhesiolysis were liver and bowel injury. Seven access related bowel injuries occurred with previous abdominal surgery and none in those with no previous surgery (0.38% vs. 0%; *p* = 0.003). Of these, five had small bowel injuries including three minor tears during adhesiolysis or retraction which were repaired laparoscopically. Two required open conversion; one with no postoperative consequences and one who had dense adhesions following total gastrectomy which resulted in a small bowel injury during open access. Even following conversion this patient had three enterotomies during division of adhesions to reach the liver. The patients had a postoperative small bowel fistula which was treated conservatively and resolved without reintervention. Of the two colonic injuries encountered one resulted from blunt dissection opening a cholecystocolic fistula which was repaired but the patient developed a postoperative collection. Laparoscopic drainage of a subphrenic collection was carried out, followed by a second intervention and defunctioning ileostomy. A second patient who had a pyloromyotomy as a child, had dense right upper quadrant omental adhesions divided using monopolar grasper and blunt swab dissection to separate the hepatic flexture from the gallbladder. Although this appeared uneventful as the adhesions did not directly involve the bowel wall and there was no sign of overt thermal spread the patient had peritonitis three days later and a thermal colonic perforation was discovered at laparotomy and a right hemicolectomy was performed. The patient succumbed to overwhelming sepsis resulting in the only death related to adhesiolysis and previous surgery in this cohort. There were no significant differences in the rates of post-operative complications, readmission, reintervention or mortality between those with previous abdominal surgery and those with no previous surgery. There were no bile duct injuries in the previous surgery cohort.

**Table 3:** Peri-operative complications and outcome parameters

Outcome	Complications (%)	All patients *n* = 5916 341 (5.76%)	Previous surgery *n* = 1846 113 (6.12%)	No previous surgery, *n* = 4070 228 (5.60%)	*p*-value (previous vs. no surgery)
Post-operative complication	Significant bleeding	19 (0.3)	5 (0.3)	14 (0.3)	0.65
	Pancreatitis	29 (0.5)	9 (0.5)	20 (0.5)	0.98
	Retained stone	28 (0.5)	6 (0.3)	22 (0.5)	0.26
	Liver injury	7 (0.1)	6 (0.3)	1 (0.0)	**0.002**
	Bowel injury	**7 (0.1)**	**7 (0.4)**	**0 (0.0)**	** < 0.001**
	Bile leak	35 (0.6)	14 (0.8)	21 (0.5)	0.26
	Collection	6 (0.1)	3 (0.2)	3 (0.1)	0.32
	CBD injury	3 (0.0)	0 (0.0)	3 (0.1)	0.56
	T-tube complication	5 (0.0)	1 (0.0)	4 (0.1)	0.59
	Chest infection	46 (0.8)	16 (0.9)	30 (0.7)	0.59
	Myocardial infarction	2 (0.0)	0 (0.0)	2 (0.0)	1
	Mesenteric ischaemia	2 (0.0)	0 (0.0)	2 (0.0)	1
	Wound infection	56 (0.9)	18 (1.0)	38 (0.9)	0.88
	UTI	2 (0.0)	0 (0.0)	2 (0.0)	1
	Dehydration	6 (0.1)	1 (0.0)	5 (0.1)	0.44
	Significant Pain	20 (0.3)	9 (0.5)	11 (0.3)	0.18
	Fever	17 (0.3)	4 (0.2)	13 (0.3)	0.49
	Vomiting	6 (0.1)	2 (0.1)	4 (0.1)	0.91
	Urinary retention	17 (0.3)	3 (0.2)	14 (0.3)	0.23
	Other	28 (0.5)	9 (0.5)	19 (0.5)	0.91
Clavien-Dindo Complications*					
Grade I		170 (2.8)	55 (3)	115 (2.8)	
Grade II		139 (2.3)	47 (2.5)	92 (2.2)	
Grade III		18 (0.3)	5 (0.27)	13 (0.3)	
Grade IV		6 (0.1)	1 (0.0)	5 (0.1)	
Grade V		8 (0.13)	5 (0.27)	3 (0.07)	
Readmissions		188 (3.2)	68 (3.6)	120 (2.9)	0.14
Reoperation due to operative complications		20 (0.3)	8 (0.4)**	12 (0.3)	0.40
Median total hospital stay		4	4	4	0.54
Mortality		8 (0.1)	5 (0.3)	3 (0.1)	0.06

Bold values indicate statistically significant

*Patients who had more than one complication were given the highest Clavien-Dindo grade

**No reoperation related to access or adhesiolysis complications

### Type of previous surgery

The most common specialty of previous abdominal operations was gynaecology (20.2%) followed by lower gastrointestinal resections (19.1%) and Obstetrics (18.5%) (Table 4). The nature of previous surgery was not recorded in 423 patients (22.9%) and although in 60% of these the site of the scar was known, mostly lower abdominal suggesting previous caesarean sections and appendicectomies, they were excluded from further analysis.

**Table 4:** Operative data and difficulty of laparoscopic cholecystectomy in patient subgroups split by type of previous abdominal surgery compared to the no previous surgery cohort

	No previous surgery (*n* = 4070)	Previous surgery, specialty					
		Hernia *n* = 49 (%)	Biliary, *n* = 24 (%)	Upper GI, *n* = 97 (%)	Lower GI, *n* = 352 (%)	Gynaecologyn = 373 (%)	Obstetric, *n* = 341 (%)
Median operative time (min)	60	70	130	90***	60	55	56
Gallbladder adhesiolysis	2561 (62.9)	36 (73.5)	12 (50)	74 (76.3)**	250 (71.0)**	233 (62.5)	211 (59.8)
Hepatic flexure adhesiolysis	800 (19.7)	10 (20.4)	16 (67)***	48 (49.5)***	94 (26.7)**	70 (18.8)	51 (14.4)*
Duodenal adhesiolysis	1839 (45.2)	27 (55.1)	22 (91.6)	72 (74.2)***	199 (56.5)***	181 (48.5)	163 (46.2)
Distal adhesiolysis	295 (7.2)	22 (44.9)***	14 (58.3)***	48 (49.5)***	108 (30.7)***	98 (26.3)***	74 (30.7)***
Calots difficult dissection	1686 (41.4)	18 (36.7)	20 (83)***	57 (58.8)***	142 (40.3)	129 (34.6)*	106 (31.1)***
Fundus-first technique	119 (2.9)	2 (4.1)	0	7 (7.2)**	12 (3.4)	6 (1.6)	8 (2.3)
Nassar difficulty grading							
I	1351 (33.2)	9 (18.4)*	0	15 (15.5)***	101 (28.7)	135 (36.2)	133 (55.2)
II	1200 (29.5)	16 (32.7)	3 (12.5)	19 (19.6)*	95 (27.0)	123 (33.0)	113 (33.1)
III	835 (20.5)	16 (32.7)*	8 (33.3)	24 (24.7)	79 (22.4)	63 (16.9)	64 (18.8)
IV	605 (14.9)	6 (12.2)	12 (50)***	31 (32.0)***	65 (18.5)	46 (13.0)	25 (7.3)
V	78 (1.9)	2 (4.1)	1 (4.2)	8 (8.2)***	12 (3.4)	6 (1.7)	6 (1.8)
Conversion to open	23 (0.6)	0 (0.0)	0 (0.0)	1 (1.0)	2 (0.6)	2 (0.5)	0 (0.0)
Abdominal drain	2120 (52.1)	31 (63.3)	15 (62.5)	69 (71.1)***	192 (54.5)	169 (47.9)	165 (48.4)

*p-value < 0.05

**p-value < 0.01

***p-value < 0.001

When patients with previous upper gastrointestinal surgery were compared to those with no previous surgery they had longer median operative time (90 vs. 60 min, *p* < 0.001), higher rates of difficult cystic pedicle dissection (58.8 vs 41.4%, *p* < 0.001), higher rates of requirement for gallbladder, duodenal, hepatic flexure and distal adhesiolysis, and fundus-first approach. These patients also had the highest difficulty grades and required more utilisation of abdominal drains.

Following previous lower gastrointestinal surgery, there were higher rates of gallbladder, duodenal, hepatic flexure and distal adhesiolysis (*p* < 0.01) but the operative difficulty grading was not significantly different to those without previous abdominal surgery.

In those with previous gynaecological or obstetric surgery, the rate of difficult cystic pedicle dissection was significantly lower than those without previous surgery. This may be related to this patient cohort being female. In both the gynaecology and obstetric subgroups there was no significant difference in rates of duodenal or gallbladder adhesiolysis, rates of fundus-first or the difficulty grading.

In surgery of all types, the rates of distal adhesiolysis were higher (*p* < 0.001). Of the five open conversions in the previous surgery group only three were caused by the presence of postoperative adhesions, two resulting in small bowel injury. No subgroup had higher rates of conversion (*p* > 0.05).

### Previous biliary surgery

Twenty four patients had undergone a biliary operation prior to referral; 8 open cholecystectomies (2 with bile duct exploration), 7 laparoscopic cholecystectomies, 4 open cholecystostomies (one with a bile duct exploration), 2 laparoscopic cholecystostomies and 3 attempted but aborted open cholecystectomies. 10 had also had previous ERCPs. 16 of these (66%) required bile duct explorations (14 choledochotomies and 2 transcystic). Subsequently, the median operative time was 130 min. The procedure difficulty was grade II in 3 patients, III in 8, IV in 12 and V in 1. Although these were more difficult, with 83% difficult cystic pedicle and higher difficulty grades than the rest of the cohort (Grades 4 and 5 in 54%) there was no fundus-first dissection, conversion, biliary complications or mortality. The median total hospital stay was 13 days on account of the inclusion of previous episodes and the fact that two thirds needed bile duct explorations, mostly via choledochotomy.

### Laparoscopic versus open previous surgery

Previous open surgery increased the operative time of the cholecystectomy compared to previous laparoscopic surgery (65 min vs. 55 min; *p* < 0.001), the rate of difficult cystic pedicle dissection (42.4% vs. 36.0%; *p* < 0.05), gallbladder, duodenal, hepatic flexure and distant adhesiolysis (*p* < 0.05) and the requirement for the fundus-first approach (4% vs 2%; *p* < 0.05). The difficulty grading was higher in those with previous open surgery than their laparoscopic counterparts. Of those who had previous upper GI surgery, the requirement for hepatic flexure adhesiolysis was higher in those with previous open versus laparoscopic surgery (*p* < 0.01) but there was no significant difference between the other parameters (Table 5).

**Table 5:** Comparison of the effects of laparoscopic vs. open previous upper and lower GI surgery on the difficulty of laparoscopic cholecystectomy

	All		Upper GI		Lower GI	
	Lap (*n* = 603)	Open (*n* = 547)	Lap (*n* = 40)	Open (*n* = 57)	Lap (*n* = 115)	Open (*n* = 237)
Median operative time (min)	55***	65***	90	92.5	60	60
Gallbladder adhesiolysis	382 (63.3)*	379 (69.3)*	29 (72.5)	45 (78.9)	84 (73.0)	166 (70.0)
Hepatic flexure adhesiolysis	114 (18.9)***	161 (29.4)***	15 (37.5)*	33 (57.9)*	34 (29.6)	60 (25.3)
Duodenal adhesiolysis	292 (48.4)**	314 (57.4)**	26 (65.0)	46 (80.7)	69 (60.0)	130 (54.9)
Distant adhesiolysis	137 (22.7)**	175 (32.0)**	20 (50.0)	28 (49.1)	46 (40.0)**	62 (26.2)**
Calots difficult dissection	217 (36.0)*	232 (42.4)*	24 (60.0)	33 (57.9)	47 (40.9)	95 (40.1)
Fundus-first technique	12 (2.0)*	22 (4.0)*	4 (10.0)	3 (5.3)	1 (0.9)	11 (4.6)
Difficulty grading						
I	214 (35.5)**	146 (26.7)**	9 (22.5)	6 (10.5)	33 (28.7)	68 (28.7)
II	197 (32.7)	152 (27.8)	7 (17.5)	12 (21.1)	29 (25.2)	66 (27.8)
III	99 (16.4)***	130 (23.8)***	9 (22.5)	15 (26.3)	24 (20.9)	55 (23.2)
IV	73 (12.1)***	105 (19.2)***	9 (22.5)	22 (38.6)	22 (19.1)	43 (18.1)
V	21 (3.5)	14 (2.6)	6 (15.0)	2 (3.5)	7 (6.1)	5 (2.1)
Abdominal drain	282 (46.8)**	299 (54.7)**	26 (65.0)	43 (75.4)	64 (55.7)	128 (54.0)

Excludes caesarean sections

*p-value < 0.05,**p-value < 0.01***p-value < 0.001 laparoscopic compared to open (all lap vs. all open; lap upper GI vs. open upper GI; lap lower GI vs. open lower GI)

### Modified access techniques

The modified access techniques used across the cohort in patients with previous surgery are reported in Table 6. Three techniques were utilised in 163 patients (2.8%) to improve access: epigastric access, supraumbilical access and access through an abdominal hernia. The most common modified access technique used was the epigastric access technique (*n* = 68) which was most commonly utilised in patients with previous lower GI surgery (*n* = 31; 45.6%). The supraumbilical technique was most utilised in patients with previous gynaecological surgery (*n* = 26; 44.8%). Of the 37 patients where hernias were utilised to aid access, there were 34 umbilical hernias, one epigastric hernia and two large incisional hernias. Epigastric access resulted in the cannula initially entering the liver parenchyma in one patient but with no postoperative consequences. There were no peri-operative complications in any case where access was achieved through a supraumbilical technique or through an abdominal hernia.

**Table 6:** Modified access techniques in different types of previous surgery

Technique	Number and percentage of previous procedures					
	Hernia	Upper GI	Lower GI	Gynae	Obstetric	Other/unknown
Epigastric (*n* = 68)	10 (14.7)	2 (2.9)	31 (45.6)	12 (17.6)	2 (2.9)	15 (22.1)
Supraumbilical (*n* = 58)	1 (1.7)	0 (0.0)	12 (20.7)	26 (44.8)	8 (13.8)	14 (24.1)
Through abdominal hernia (*n* = 37)	0 (0.0)	4 (10.8)	7 (18.9)	10 (27.0)	3 (8.1)	14 (37.8)

## Discussion

To our knowledge, this is the largest cohort study to investigate the relationship between the outcomes of cholecystectomy in patients with previous surgery, the various types of procedures and the use of modified access techniques. The study showed that previous surgery, regardless of the extent and the location of previous scars, is far from a contraindication to laparoscopic cholecystectomy. Although some reports suggested that certain types of previous abdominal surgery could be considered a relative contraindication, this cohort had no exclusions from laparoscopic surgery based on the presence of scars of previous operations. Only three of five open conversions were associated with the need for adhesiolysis and one of these was the result of bowel injury during access.

The utilisation of modified access techniques for cholecystectomy in patients who had previous surgery has not been adequately addressed in relation to specific operations or scar location. This report describes a number of techniques that can be employed to reduce the risk of access complications, mainly by selecting to place the initial camera ports by open access and away from any abdominal scars.

Previous abdominal surgery, particularly biliary and upper gastrointestinal surgery as well as open procedures increased the difficulty of the laparoscopic cholecystectomy due to the need to divide postoperative adhesions and this relationship is well established [12–17]. Postoperative adhesions can result from cauterisation, suturing and any damage to tissue surfaces producing ischaemia, disrupting lymphatic drainage or preventing revascularisation. They are extremely common following major abdominal surgery (90%) and occur in roughly 55–100% of patients undergoing pelvic surgery [16].

In this study the requirement for separating adhesions between the gallbladder and either the duodenum (RR = 1.07; *p* = 0.023) or the hepatic flexure (RR 1.11; *p* = 0.043) increased slightly in patients who had previous surgery. This would be expected to occur in patients who had undergone upper abdominal surgery. However, although such adhesions to the gallbladder are also encountered in patients who had previous cholecystitis or pancreatitis without abdominal scars, the need to divide distant adhesions was significantly associated with previous surgery (RR 3.57; < 0.001).

When considering the whole cohort of patients who had scars of previous abdominal operations, there were no significant differences in the difficulty grading of the cholecystectomy, the use of alternative surgical approaches (e.g. conversion or fundus-first) or the rate of utilising abdominal drains in those with previous surgery compared to those with no previous surgical history (Table 2). The rate of abdominal drainage was relatively high in both groups, just over 50%, due to the unit’s high incidence of emergency admissions and the interest in bile duct exploration.

It seems that previous upper abdominal surgery presents a different picture. The impact of previous surgery and adhesions on the difficulty of cholecystectomy has already been investigated by Lee et al. who performed propensity score matching between 117 patients [4]. They found that patients who underwent previous upper abdominal surgery had longer operative time, higher conversion rates, complication rates and prolonged hospital stay. Despite a different method of analysis, the current study arrived at a similar conclusion and more specifically demonstrated that previous upper gastrointestinal surgery and previous open surgery were more significantly associated with adhesion formation, longer operation time, difficult cystic pedicle dissection and higher cholecystectomy difficulty grading.

Karayiannakis’ cohort of 1638 laparoscopic cholecystectomies similarly found that in the 473 patients with previous abdominal surgery there were longer operation time, higher rates of conversion and a higher incidence of post-operative wound infection [5]. However, the present study did not show increased rates of open conversion in such patients. This may be due to the subspecialist interest of the senior author who performed most cholecystectomies in the institution and received referrals of patients with complex biliary conditions from other hospitals. The use of modified access techniques reduced the rate of complications, such as bowel injury, which would typically result in the need for conversion. Our study suggests, therefore, that previous surgery does not increase peri-operative morbidity through higher conversion rates [18, 19], perhaps because of the use of modified access techniques.

Katar et al. found that the rate of operative complications in patients with previous abdominal surgery undergoing laparoscopic cholecystectomy was no different to those without [3]. This is in contrast to the current study where the risk of liver and bowel injury, although small, was significantly higher in those with previous surgery (0.3% vs. 0.0%; *p* < 0.003). However, this is the result of the much larger sample size in this study allowing the recording and the identification of significance of such findings. Although the risk of bowel injury can be mitigated by open access methods at a remote site avoiding the scars, modified access methods are probably unlikely to completely eradicate bowel injury. Releasing small bowel adhesions was only carried out if absolutely necessary and alternative camera port sites were chosen to avoid bowel any adherent to the parietal peritoneum behind the umbilicus. However, 6 of 7 such injuries occurred during adhesiolysis and only one during access.

Whilst there is a strong association between previous surgery, particularly upper gastrointestinal and biliary surgery, and increased difficulty of laparoscopic cholecystectomy, a broader perspective should be considered during pre-operative assessment. Previous abdominal surgery is far from being the sole determinant of intra-abdominal adhesions, the requirement for adhesiolysis or the difficulty of the laparoscopic cholecystectomy. Multiple other studies have identified factors contributing to difficult cholecystectomy (previous cholecystitis, pancreatitis and ERCP), often with much higher odds ratios than those of previous abdominal surgery alone [20, 21]. All of these factors should also be borne in mind during patient assessment prior to laparoscopic cholecystectomy.

The details of the previous surgery, particularly open versus laparoscopic approach and the speciality of operation should be determined and carefully considered in the surgical planning of access methods and in assessing the potential for significant adhesions. The distinction between patients who had previous upper and lower abdominal surgery has been highlighted, comparing the outcomes [22]. However, the relevance of the speciality of the procedure to the resulting scarring and adhesions was less well defined.

To our knowledge this study is the first to investigate the relationship between an objective classification of the difficulty of cholecystectomy and the outcomes of surgery in the presence of previous scars resulting from such a range of operations. It is important to note that previous gynaecological, obstetric and hernia surgery do not present the same degree of risk as previous upper and lower gastrointestinal surgery or biliary surgery. The type of previous surgery in such cases does clearly influence the intra-operative difficulty grade encountered (e.g. requirement for adhesiolysis and difficult Calot’s dissection).

The use of optical trocars was described where peritoneal entry is achieved step by step under visual guidance. String et al. described the routine use of optical trocars to create a pneumoperitoneum in 650 laparoscopic procedures. Although they did not specifically address cholecystectomy they report a complication rate of 1.2% in patients with previous abdominal surgery [23]. Mohammadi et al. compared optical trocars to open access techniques and reported a higher complication rate with optical trocars (8% vs. 0%), although their cohort did not exclusively include patients with previous abdominal surgery [24]. Although the outcomes of optical techniques have not been formally investigated in patients with previous surgery undergoing cholecystectomy, similar studies suggest that the modified access techniques described in this cohort have superior outcomes [25]. This unit has had no experience with using optical trocars.

The use of the blind approach, using a needle to establish pneumoperitoneum and to insert the first camera port was described by Lécuru et al. [26]. Although their study only included patients who had this blind access method employed through the umbilicus, regardless of the presence of abdominal scars, they recommended a “search” for alternative approaches in such patients. They reported a complication rate of 2.6% in patients undergoing laparoscopy with previous laparotomies. Although they recognised the importance of adapting the access method in patients with abdominal scars their series was retrospective and involved an unexplained, and probably unjustifiable, use of the blind approach throughout. On the other hand, the use of the needle technique in the first part of the current study was modified in patients with abdominal scars by inserting the needle on the side of the umbilicus opposite a scar, through an umbilical hernia defect or inserting a lateral port first. The open access technique was also occasionally used.

Prieto-Díaz-Chávez et al. [27] compared direct trocar insertion without pneumoperitoneum being pre-induced using Verres needles in laparoscopic cholecystectomy. They reported a complication rate of 2.3% for direct trocar insertion vs. 23.8% for routine use of Verres needle during laparoscopic cholecystectomy irrespective of previous surgery. The Verres needle complication rate was inexplicably higher than in other series and, therefore, the authors’ conclusion was understandably biased towards their preferred direct trocar insertion which had significantly lower morbidity. The high rate of complications in Pietro-Dias-Chaves is in clear contradiction to the results of the early part of the current study, where no access related complications occurred using the Verres needle method. Direct trocar entry was never used on this unit.

Direct optical entry using a 5 mm telescope through a 5 mm trocar at Palmer’s point was also proposed as a safe method of establishing pneumoperitoneum [28]. However, should an injury occur to omentum or bowel with a Verres needle or a direct entry the surgeon is blinded to it. A small or large bowel segment which is adherent underneath the access site can easily be entered, increasing the risks of repeated attempts due to difficulty in establishing pneumoperitoneum and unsuccessful entry. An injury caused by a Verres needle may be compounded by the subsequent blind insertion of a trocar. A surgeon would still be left with the need to establish proper access in order to inspect the area and manage a potential injury laparoscopically or by open surgery. If, in the case of failed access, another site is used then the peritoneal aspect of the original site must be cleared and inspected to avoid missing an injured viscus which can result in serious morbidity.

In our practice open subxiphoid access never resulted in bowel injury as bowel adhesions are less likely to form due to the presence of the falciform ligament. Repeating and redirecting cannula insertion and using optical guidance are both possible. This caused only one complication with the cannula initially entering the liver parenchyma with no operative or postoperative consequences.

### Limitations

The significance of previous surgery, abdominal scars and the benefits of modified-position open access techniques must be seen in the light of this unit’s specialist interest in laparoscopic biliary surgery and a high workload of emergency biliary admissions. While the results may not entirely reflect the practice of the general surgeon performing cholecystectomies less frequently, the access techniques described are simple and applicable irrespective of expertise.

However, surgeons should carefully consider the implications of abdominal scars, their ability to establish safe pneumoperitoneum through modifying their techniques for access and adhesiolysis and whether the involvement of, or referral to, a more experienced surgeon is appropriate in selected patients.

Future research should perhaps compare series of cholecystectomies performed on patients with abdominal scars to confirm the trends reported by this study. It will be interesting to demonstrate a relationship between the workload and the outcome parameters for such patients and more importantly the value of specialisation in benign biliary surgery.

## Conclusion

This study demonstrates the significance of the specific type of previous abdominal operations and the location of their scars in a large cohort of patients undergoing laparoscopic cholecystectomy with detailed prospective tracking of access modifications and operative and postoperative outcomes. The insertion of initial access ports should be guided by the position of the abdominal scars. Supraumbilical access avoids suprapubic or lower midline scars of gynecological procedures not reaching the umbilicus while epigastric access is used in patients with lower midline or paramedian scars of colonic surgery extending above the umbilicus and those with colostomies or ileostomies. Abdominal wall hernias offer safe access when used across a range of previous surgery types. This study, the largest addressing laparoscopic access in patients with scars of a range of previous abdominal procedures, demonstrates the importance of tailoring access techniques to the scar location and to the type of the previous procedure in individual patients. It also highlights the benefits of specialisation.
